# Long-Term Changes in Cyanobacteria Populations in Lake Kinneret (Sea of Galilee), Israel: An Eco-Physiological Outlook

**DOI:** 10.3390/life5010418

**Published:** 2015-02-05

**Authors:** Ora Hadas, Aaron Kaplan, Assaf Sukenik

**Affiliations:** 1 The Yigal Allon Kinneret Limnological Laboratory, Israel Oceanographic and Limnological Research, Migdal, Israel; 2 Department of Plant and Environmental Sciences, The Hebrew University of Jerusalem, Jerusalem, Israel; E-Mail: aaron.kaplan@mail.huji.ac.il; 3 The Yigal Allon Kinneret Limnological Laboratory, Israel Oceanographic and Limnological Research, Migdal, Israel; E-Mail: assaf@ocean.org.il

**Keywords:** Lake Kinneret, phytoplankton shift, cyanobacteria, nutrients, management

## Abstract

The long-term record of cyanobacteria abundance in Lake Kinneret (Sea of Galilee), Israel, demonstrates changes in cyanobacteria abundance and composition in the last five decades. New invasive species of the order Nostocales (*Aphanizomenon ovalisporum* and *Cylindrospermopsis raciborskii*) became part of the annual phytoplankton assemblage during summer-autumn. Concomitantly, bloom events of *Microcystis* sp. (Chroococcales) during winter-spring intensified. These changes in cyanobacteria pattern may be partly attributed to the management policy in Lake Kinneret’s vicinity and watershed aimed to reduce effluent discharge to the lake and partly to climate changes in the region; *i.e.*, increased water column temperature, less wind and reduced precipitation. The gradual decrease in the concentration of total and dissolved phosphorus and total and dissolved nitrogen and an increase in alkalinity, pH and salinity, combined with the physiological features of cyanobacteria, probably contributed to the success of cyanobacteria. The data presented here indicate that the trend of the continuous decline of nutrients may not be sufficient to reduce and to control the abundance and proliferation of toxic and non-toxic cyanobacteria.

## 1. Introduction

Cyanobacteria are found in a diverse range of habitats, from oceans to freshwater, from bare rock to soil, from hot springs and hydrothermal vents to the arctic and to areas under ice. There are growing ecological, environmental and health concerns worldwide regarding the expansion of toxic and harmful cyanobacterial blooms that cause deterioration in water quality, generate anoxia and alter or disrupt existing food webs in marine and freshwater environments. Bloom-forming genera, some of which produce toxins, for example *Microcystis*, *Anabaena*, *Aphanizomenon* and *Cylindrospermopsis*, often dominate the photoautotrophic community of these types of water bodies [1]. In addition, toxic secondary metabolites (*i.e.*, microcystins, cylindrospermopsin and nodularin) produced by many cyanobacterial species pose a serious threat for humans and livestock. Cyanobacterial blooms are attributed to the nutrient enrichment of waters (eutrophication) by urban, agricultural and industrial development [2]. Recently, it was suggested that the increase in *Microcystis* bloom events might be connected to climate change, but the contribution of major nutrients (N and P) should not be ignored [3,4]. Blooming events of cyanobacteria of the order Nostocales (mainly the genera *Aphanizomenon* and *Cylindrospermopsis*) have intensified over the last decade in many freshwater lakes and reservoirs worldwide [5,6,7,8,9,10,11]. Their appearance and blooms may point to global climate change [12].

Reduced nutrient loading into lakes resulted in a shift in the phytoplankton composition [13], but in some cases, despite the reduction in nutrients, higher chlorophyll-*a* was measured [14]. Dokulil and Teubner [15] found that in Lake Mondsee, Austria, although a decline in total phosphorus (TP) was observed, the phytoplankton biovolume continued to increase, suggesting that there is a time lag in the response of various phytoplankton species to the decrease in nutrient concentrations.

In the present chapter, we describe a case study that demonstrates the dynamics of the cyanobacteria population in the warm monomictic Lake Kinneret, Israel, also known as the Sea of Galilee, over half a century. Using a long-term record of biotic and abiotic parameters, we further evaluate the role of regional and global changes in controlling the temporal/seasonal and multiannual variations in the abundance of cyanobacteria populations and species composition. In addition, the physiological responses of various bloom-forming species isolated from Lake Kinneret (*A. ovalisporum* and *C. raciborskii* of the order Nostocales; *Microcystis* sp. of the order Chroococcales) were studied to elucidate eco-physiological conditions that supported the appearance, proliferation and establishment of cyanobacteria in the lake.

## 2. Study Site

Lake Kinneret, located in Israel (32°42'–32°55'N; 35°31'–35°39'E), is a warm monomictic freshwater lake, with a surface area of 170 km^2^, a total water volume of 4 × 10^9^ m^3^ and a mean and maximum depth of 25 and 42 m, respectively. The lake is stratified for eight months, from April to December, every year, forming an aerobic warm (24–30 °C) epilimnion and an anoxic colder (14–16 °C) hypolimnion, with high sulfide concentrations (~5.0 to 10 mg L^−1^) and ammonium (~0.5 to 1.3 mg L^−1^ N-NH_4_). Winter floods are the main sources of dissolved inorganic nitrogen (DIN), providing about 75% in the form of NO_3_. In addition, NH_4_ from the hypolimnion and sediments is distributed through the water column upon thermal de-stratification (winter turnover), reaching a concentration of 600 µg L^−1^. DIN sources in the summer (between June and October) are limited, resulting in low DIN concentration in the epilimnion. Soluble reactive phosphorus (SRP) concentrations in the epilimnetic water column are low, in the range of 1 to 5 µg P L^−1^, with a maximum of 10 µg P L^−1^ during overturn [16]. The yearly average Secchi ranged between 2.3 and 4 m in depth.

## 3. The Players

**Figure 1:**
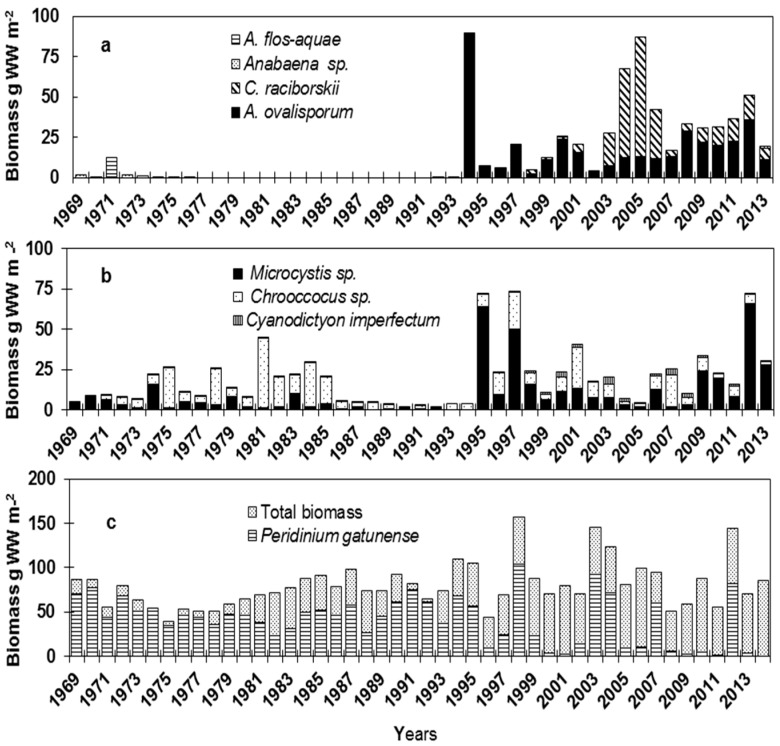
The annual maximum biomass of (a) Nostocales species, (b) Chroococcales species and (c) *Peridinium* and total biomass in Lake Kinneret, 1969–2014. The biomass measurements were based on phytoplankton counts in samples collected from various depths at biweekly intervals. Depth-integrated biomass was done as described by Berman and Pollingher (1974) [17] and Zohary (2004) [18]. During stratification, the depth-integrated biomass was calculated from zero to mid-thermocline, between 15 and 20 m (epilimnion); during mixis, the integration was over the entire water column. Wet weight (WW) was calculated according to the geometric shape of the species [19], assuming that the density of the algae is one.

The long-term record of cyanobacteria in Lake Kinneret comprises 41 species and genera from three main orders; Nostocales, Chroococcales and Oscillatoriales. Members of the latter order were sporadically observed [20]. Variations in the relative species composition and domination on multiannual scales (Figure 1) demonstrate that the annual total cyanobacteria biomass can be divided into three periods: (1) from 1969 to the mid-1980s; (2) from the mid-1980s to the mid-1990s; and (3) from 1994 to present. During the first period, cyanobacteria comprised a small fraction of the total phytoplankton biomass (the annual average from 1969–1985 ranged between <0.1% and 0.9% of the total biomass), and Chroococcales species dominated the cyanobacterial assemblage. From the mid-1980s to the mid-1990s, the cyanobacteria population was substantially suppressed. During that period, the dominating taxa were *P. gatunense* during winter-spring. *Aulacoseira granulata*, which reached a biomass of 182 g m^−2^ in 1988, and chlorophytes (*Pediastrum* sp., *Coelastrum* sp., *Scenedesmus* sp. and *Tetraedron*). Since 1994, blooms of *Microcystis* (Chroococcales) and blooms of *Aphanizomenon ovalisporum* and *Cylindrospermopsis raciborskii* (Nostocales) intensified [11,18,21,22]. Here, we bring a short description of the common and most abundant cyanobacteria species observed in Lake Kinneret.

### 3.1. Nostocales

A minor appearance of Nostocales was recorded in Lake Kinneret during the early 1970s, with *Aphanizomenon flos-aquae* and *Anabaena* sp. reaching maximum biomass values of 11.9 and 1.4 g m^−2^ in 1971 and 1972, respectively (Figure 1). No Nostocales were recorded in the lake from 1977–1992. Perennial blooms of Nostocales emerged with the appearance of *A. ovalisporum* and *C.* *raciborskii* in the autumn of 1994 and in the summer of 1998, respectively. These two species had appeared each summer, albeit at varying abundance. The maximum biomass of about 89 and 74 g wet weight (WW) m^−2^ for *A. ovalisporum* (in 1994) and *C. raciborskii* (in August, 2005), respectively, was recorded, contributing about 80% of the phytoplankton biomass [23,24,25]. The *C. raciborskii* bloom in summer 2005 collapsed in early September, followed by a much lower peak of the *A. ovalisporum* population in November. The long-term record (Figure 1) suggests two alternating periods since the 1990s, where *A. ovalisporum* dominated the summer-autumn population between 1994 and 2002 and again since 2007, whereas *C. raciborskii* was conspicuous between 2003 and 2006 [22]. The repetitive appearance of *A. ovalisporum* in Lake Kinneret, as well as in European lakes [26,27] suggests that the species is expanding its geographic range similarly to *C. raciborskii*, presumably due to regional and global changes that meet its physiological demands [11,12].

### 3.2. Chroococcales

The main cyanobacteria species reported in Lake Kinneret before 1994 were *Microcystis* sp. (*Microcystis aeruginosa*, *Microcystis wesenbergii*, *Microcystis botrys* and *Microcystis flos-aquae*), *Cyanodictyon imperfectum* and *Chroococcus minutus*. *C. minutus* was the main contributor to cyanobacteria biomass during 1973–1989 (Figure 1). Large fluctuations in the abundance of *C. minutus* were attributed to the availability of nutrients and to grazing pressure by herbivorous zooplankton, with a maximal biomass equaling 10% of the total phytoplankton biomass recorded in 1984 [23]. *C. minutus* appeared in the lake during summer (June–September). Other species of *Chroococcus* (*C. limneticus* and *C. turgidus*) appeared sporadically. *Cyanodictyon imperfectum* was present in January–February and disappeared in April–June due to shading by the annual characteristic winter-spring bloom of the dinoflagellate, *Peridinium gatunense*, because of competition for nutrients. It reappeared and reached maximum abundance during summer-autumn [28]. *Microcystis* sp. appears in the lake after overturn during the mixing period in January–March, preceding the *Peridinium gatunense* bloom and, during some years, competing with *Peridinium*, depending on weather conditions and turnover time [29]. Until 1994, only occasional winter bloom events of *Microcystis* sp. were recorded (in 1964, 1971, 1976 and 1983), but since 1994, blooms of *Microcystis* sp. have been common in the lake and delayed the development of *Peridinium*, probably due to allelopathic interactions between the two species [30,31]. *Microcystis aeruginosa* was the dominant Chroococcales species in 1995, 1997 and 2012, with massive blooms reaching values of 64, 50 and 66 g WW m^−2^, respectively (Figure 1).

### 3.3. Total and Peridinium gatunense Biomass 

The yearly average of total biomass until 1980 consisted mostly of *Peridinium,* contributing more than 90% to the total biomass. Since 1986, fluctuations in total and *Peridinium* biomass were recorded due to years with no *Peridinium* (*i.e.*, 1996, 2000, 2001 and 2008; Figure 1c) and/or years with intensified blooms of *Peridinium* (*i.e.*, 1998, 2003 and 2012; Figure 1c). In addition, the contribution of *Peridinium* to the total biomass reduced (25%–65%), and other species, mainly cyanobacteria (*A. ovalisporum, C. raciborskii*), but also *Mougeotia* sp., contributed to the increased total biomass (Figure 1c). Thus, although there was a reduction in total nitrogen (TN), TP, total dissolved nitrogen (TDN) and total dissolved phosphorus (TDP) concentrations in Lake Kinneret, the total biomass increased, with a shift in species composition, as was also found for Lake Mondsee, Austria [15]. Global warming and an increase of the water column temperature may also support the phytoplankton shift [13,22].

## 4. Long-Term Variations in Environmental Conditions

The appearance and blooms of cyanobacteria in the lake during the last two decades, compared to their low abundance during the earlier period, raised the question: what gave the members of this group the advantage in proliferating and establishing themselves in the lake? The long-term data of Lake Kinneret [32] show that the ambient conditions in the lake have changed in recent decades. These changes, which may be ascribed to global and regional climate change, include a slight increase in the air temperature during spring and summer, less precipitation, reduced water inflow and changes in the stratification pattern [33,34,35]. Anthropogenic interventions in the lake and in its watershed were applied in order to provide a wide range of services for the local population. Since 1964, the national water carrier has been operated for both domestic use and irrigation. Consequently, over-pumping resulted in multiannual water level fluctuations beyond its natural span. In the 1980s, recycled domestic (sewage) and agricultural (cowshed) effluents were diverted from the watershed to nearby water reservoirs for local agricultural reuse. For various agricultural and hydrological reasons, a restricted area of the Hula Valley (located in the watershed and responsible for draining much of the incoming water) was re-flooded in 1994, together with the employment of revised agricultural practices [36].

These regional changes enforced temporal variations in water inflow and in nutrient and solid loads conveyed to the lake, which affected the in-lake nutrient status, with a primary impact on the phytoplankton assembly.

### Temperature and Wind

The temperature record for Lake Kinneret’s epilimnion revealed an increase of ~1 °C from 1969 to 2009 [34]. Epilimnion temperatures of 29 °C and above were common in the upper water layer between 1994 and 2013, but such high temperatures were rarely observed prior to 1994 [22]. The higher frequency of these extreme temperature events imposed stronger stratification in recent years [33].

During the summer time of the 1990s, the frequency of high wind speed events (velocities of 12 m s^−1^) was 40% lower than was recorded in Lake Kinneret during the 1970s. These changes are in accordance with regional trends described in detail by Saaroni *et al.* [37], who reported a reduction in the frequency of peak wind speed events in the East Mediterranean from the 1970s to the present. These elevated temperatures and low speed wind events are favorable for cyanobacteria.

## 5. Variations in Nutrients Status

### 5.1. Total Nitrogen and Total Dissolved Nitrogen

**Figure 2:**
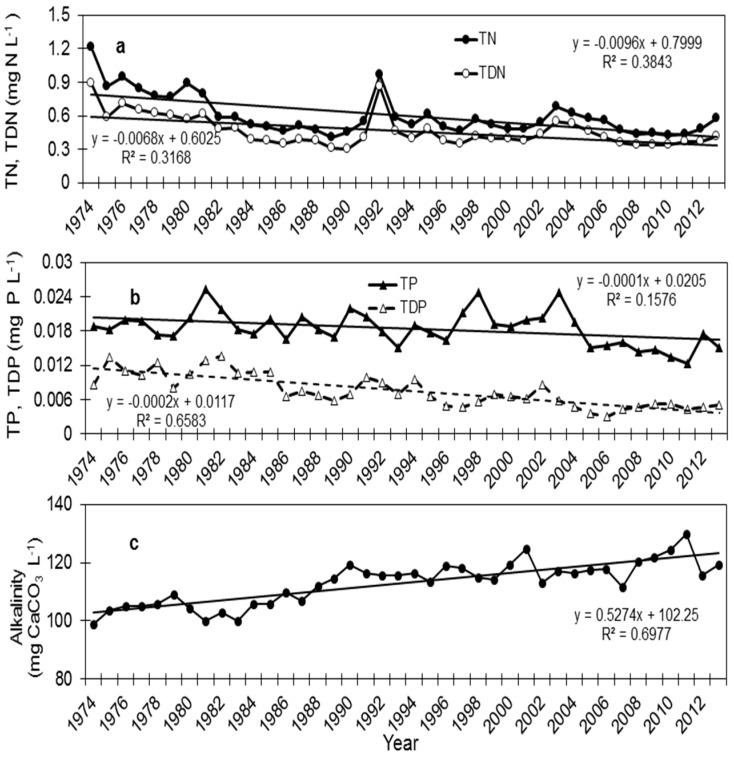
The yearly average of various limnological parameters in the upper water column of Lake Kinneret for 1974–2013: (**a**) total nitrogen (TN) and total dissolved nitrogen (TDN); (**b**) total phosphorus (TP) and total dissolved phosphorus (TDP); and (**c**) alkalinity.

The multiannual data of the yearly average of TN and TDN concentrations (Figure 2) reflect the changes in the management policy in the lake vicinity. The data can be divided into two periods: 1974–1990 and 1990–2013. The first period is characterized by a gradual decrease in TDN from 0.9 mg N L^−1^ measured in 1974 to 0.3 mg N L^−1^ measured in 1990. The second period since the 1990s began with the exceptionally rainy year of 1992 (beginning in December 1991), with an occasional increase in TP and TDN due to the water runoff entering the lake via the Jordan River. The sharp decrease in TDN, 0.39 mg N L^−1^ in 1994, continues the trend of lower TDN concentrations, with some fluctuations during exceptionally rainy years (*i.e.*, 2002–2003). A statistical analysis of the DIN (NH_4_^+^, NO_3_^−^) concentrations on an annual and seasonal basis showed a significant difference between these two periods, mostly apparent during the summer months. Much lower DIN concentrations were reported in the last two decades compared to the previous period [22]. Taking into account that concentrations of dissolved nutrients are strongly influenced by the uptake of phytoplankton, the total nitrogen (TN) concentrations depicted in Figure 2 indicate a generally multiannual trend that follows TDN. The total nitrogen ranged between 1.21 to 0.38 mg N L^−1^ in the time period from1974 to 2014 (Figure 2). The average for the years 1974–1980 was 0.9 mg N L^−1^, and since 1981, a decrease in TN was recorded with an average of 0.5 mg N L^−1^ during 2001–2014.

### 5.2. Total Phosphorus and Total Dissolved Phosphorus

The yearly average TP concentrations decreased in the upper epilimnion in the last four decades (Figure 2). During the 1970s and until the mid-1980s, the yearly average fluctuated between 17 and 25 µg P L^−1^. During the last decade, TP concentrations ranged between 12 and 17 µg P L^−1^. The long-term record indicates a gradual reduction in TP concentrations at an annual rate of 0.1 µg P L^−1^. The highest TP concentrations were observed during years of exceptionally massive blooms of the dinoflagellate, *Peridinium gatunense* (1998 and 2003; Figure 2). The yearly average TDP concentrations decreased in the upper 5 m of the epilimnion in the last four decades (Figure 2). During the 1970s and until the mid-1980s, the yearly average fluctuated between 8 and 13 µg P L^−1^. During the last decade, TDP concentrations ranged between 3 and 8 µg P L^−1^. The long-term record indicates a gradual reduction in TDP concentrations at an annual rate of 0.2 µg P L^−1^. This reduction reflects the changes of anthropogenic intervention in the watershed and is associated with the changes in the phytoplankton assemblage and the biological activity in the lake (Figure 2). However, in 1994, there was an increase in TDP values in summer-autumn, as reported by Hadas *et al.* [22], that boosted the summer bloom of *Aphanizomenon ovalisporum* (Figure 1).

### 5.3. Salinity

The in-lake salinity is balanced by water and saline inflows and outflows, evaporation and rainfall and fluctuates on a seasonal timescale [38]. The freshwater Lake Kinneret has a relatively high NaCl concentration, about ten-times higher than its main water contributor, the Jordan River. The sources of this excess salinity are offshore and near shore saline springs. From the 1950s to 2000, salinity (expressed as Cl^−^) in the lake dropped from 390 to 230 mg L^−1^, following the diversion of northwestern shoreline saline springs to the saline water carrier (SWC) in January, 1965. Since the 1990s, due to a decrease in the annual water inflows into the lake, there has been a gradual increase in water salinity, with predicted values of 470 mg Cl^−^ L^−1^ in 2040 [39]. The increase in Cl^−^ is concomitant with the increase in Na^+^ concentration that was favorable for the development of cyanobacteria of the order Nostocales, *i.e.*, *A. ovalisporum*, in the lake [40].

### 5.4. Alkalinity, pH

Fluctuations in alkalinity are observed in the lake on a seasonal scale because of the biota activity (photosynthesis, respiration). An increase in alkalinity was recorded during the last 40 years, reaching yearly average values of 130 mg CaCO_3_ L^−1^ in 2011 compared to 99 mg CaCO_3_ L^−1^ in 1974 (Figure 2c). The annual spring bloom of *P. gatunense* that consumed CO_2_ via photosynthesis imposed high pH and precipitation of calcite [41], which affected the seasonal variation in alkalinity. The high alkalinity and elevated pH supported the proliferation of cyanobacteria that can assimilate bicarbonate in addition to CO_2_ [42].

## 6. Physiological Response of Cyanobacteria Populations to the Changing Environment

The long-term changes in many environmental variables coincided with trends in the abundance of cyanobacteria. The ambient variables demonstrated a gradual and moderate increase in water column temperature, salinity and alkalinity and a gradual decrease in DIN and TDP concentrations, with occasional intrusions of phosphate; whereas, the cyanobacteria community displayed at once a prompt change in its abundance. This rapid increase in the abundance of cyanobacteria in Lake Kinneret suggests that a certain set of environmental conditions reached a “threshold” above which the proliferation of cyanobacteria was feasible. The appearance and establishment of Nostocales species since 1994 was supported by this new set of environmental conditions and was maintained by their capability to fix atmospheric nitrogen under N limitation [22] and the ability to survive unfavorable conditions by forming dormant cells, akinetes. As the result of the gradual shift in nutrient loads (decrease in N load) to Lake Kinneret, Gophen *et al.* [43] suggested that nitrogen-fixing cyanobacteria blooms might occur. This prediction came true with the summer bloom of *A. ovalisporum* in 1994. The Nostocales in summer-autumn provided as much as 123 tons of “new N” per year via nitrogen fixation, equaling up to 150% of the summer DIN (dissolved inorganic nitrogen) load [22]. Similarly, from 1995 on, an increase in Chroococcales biomass, mainly *Microcystis* sp., was observed (Figure 1).

The increase in alkalinity (Figure 2) changed the equilibrium of the inorganic carbon species in the lake and probably created an opportunity for bicarbonate users, such as Nostocales species, and a disadvantage for CO_2_ users only. In laboratory experiments, the photosynthetic half saturation constant K_1/2_ (CO_2_) for *A. ovalisporum* was 2.2 µM at pH 7.0 and declined to 0.04 µM at pH 9.0. The photosynthetic half saturation constant K_1/2_ (HCO_3_^−^) was 10 µM at pH 7.0, but increased until 24 µM at pH 9.0, indicating that *A. ovalisporum* is a bicarbonate user [44]. Similar observations were reported for *C. raciborskii* [45]. The fact that *A. ovalisporum* and *C. raciborskii* primarily utilize HCO_3_^−^ can explain the high photosynthetic rates during the bloom, under high pH and low dissolved CO_2_ conditions, as was described for other cyanobacteria species [40,42].

As lake water salinity increased over the last five decades, the Na^+^ concentrations increased from 4 to 5.4 mM, a range essential for *A. ovalisporum* growth at high pHs. These can serve as an adaptive feature of Nostocales to form summer blooms in the lake.

Lake Kinneret is considered a P-limited system, particularly during strong thermal stratification in the summer and autumn [29]. The gradual decrease in the concentrations of total dissolved phosphorus (Figure 2) restricted the phytoplankton community. Nevertheless, intrusions of phosphate from internal and external sources may support an increase in cyanobacteria abundance. Floods, water runoff, decomposition of algal blooms and dust precipitation are potential sources that may temporarily relieve the P limitation. For example, during the summer of 1994, a significant rise in the monthly average of TDP concentration was observed, due to the decomposition of an unusually massive bloom of *Peridinium gatunense*, which probably boosted the *A. ovalisporum* bloom [24]. Upon inorganic phosphorus (Pi) intrusion, *A. ovalisporum* sequestered significant amounts of Pi, using its capacity for rapid luxury uptake and the storage of Pi in polyphosphate bodies. As the external Pi concentration became limiting, Pi acquisition and storage supported about five doublings of *A. ovalisporum* and could be the reason for the proliferation and bloom of *A. ovalisporum* in Lake Kinneret in 1994. The *Aphanizomenon* blooms of 2009 and 2010 support this notion, showing that P limitation at the beginning of 2010 led to the collapse of the bloom early in the season [46]. Biotic interactions may be an additional mechanism to cope with Pi limitation in *A. ovalisporum*, as described by Bar-Yosef *et al.* [47].

Laboratory experiments on isolated cultures showed that the optimal temperature for *A. ovalisporum* and *C. raciborskii* growth ranged between 26 and 32 °C, corresponding to the occurrence of temperatures above 29 °C in the upper water column in Lake Kinneret during the last two decades [22]. *Microcystis* sp. were part of the phytoplankton community in winter-spring (temperatures of 16 to 22 °C), when nutrients were available. The higher abundance of *Microcystis* in the last two decades cannot be explained by temperature, and therefore, other factors are probably involved, as described below.

## 7. Biotic Interactions

The distribution and dominance of algal populations are affected not only by ambient variables (nutrients, temperature, light), but also by biological factors, *i.e.*, interspecies interactions, such as competition, stimulation, allelopathy and top down control (grazing). The appearance of Nostocales in the lake since 1994 and the failure of *Peridinium* to bloom in some of the years after 1994 suggest that interspecies interactions were involved in algal development and dominance in the lake. *Aphanizomenon* growth was enhanced when a filtrate taken from a senescent *Peridinium* culture was added to *Aphanizomenon* cultures; *i.e.*, degradation products of *Peridinium* present in the filtrate stimulated *Aphanizomenon* growth [24]. *Peridinium* growth was inhibited by *Microcystis* sp., and the inhibitory effect of *Microcystis* was attributed to the excretion of allelopathic substances, rather than to a successful competition for nutrients. Lyophilized *Microcystis*-free spent medium (MFSM) inhibited the steady-state photosynthesis of *Peridinium* via a specific mechanism that slowed the hydration rate of CO_2_ and the depression of the internal carbonic anhydrase activity [30]. Conversely, the application of spent *Peridinium* medium induced the sedimentation and lysis of *Microcystis* cells concomitant with a rise in mcyB, involved in toxin biosynthesis in *Microcystis*, and the response was dependent on *Peridinium* cell densities [31].

A unique mechanism for P scavenging to cope with Pi limitation in *A. ovalisporum* was described as the “enslavement” of photosynthetic eukaryotes, to provide phosphate via the recruiting of other organism’s alkaline phosphatase (APase) [39]. *A. ovalisporum* excretes secondary metabolites, such as the toxic alkaloid, cylindrospermopsin, which induces the synthesis and excretion of alkaline phosphatase in eukaryotic algae. *A. ovalisporum* with highly efficient Pi transporter (pstS) assimilate inorganic P released by this enzymatic activity. The induction of APase occurred in *A. ovalisporum* only when internal P sources were diminished. This unique mechanism provides an additional advantage to *A. ovalisporum* in its survival and domination in nutrient-depleted environments.

*Cylindrospermopsis* outcompetes *Aphanizomenon* at the beginning of the summer, due to a higher affinity to combined nitrogen. At the end of the summer and in the autumn, *Aphanizomenon* takes over, due to its more efficient nitrogen fixation capabilities [22].

Filamentous and colonial cyanobacteria are difficult to ingest due to their size and shape. Ciliates and other protozoa and small zooplankters preferentially consume single-cell coccoid cyanobacteria, resulting in the relative abundance increase of colonial and filamentous cyanobacteria. Furthermore, protozoa grazing on solitary cells could induce colony formation in *Microcystis aeruginosa* [48].

## 8. Conclusions

Lake Kinneret, as a complex aquatic ecosystem, responded to long time scale variations in environmental conditions, in multifaceted ways. The simplified description of the linear changes in environmental conditions, as described above, does not demonstrate extreme events, such as rainy or droughty years, exceptionally warm summers, *etc.*, thereby masking the possible biological responses. From a perspective of almost five decades, it is evident that Lake Kinneret is experiencing a gradual, but steady process of decline in the concentration of major macronutrients, which may lead toward oligotrophication. This process stems from a management effort that aimed to maintain high water quality. The average value of the trophic state index (TSI) for Lake Kinneret using the equation of Deng *et al.* (2014) [49] was 43, suggesting a mesotrophic toward oligotrophic status of the lake. One unpredicted outcome of such efforts was the invasion of new cyanobacterial species, *i.e.*, *A. ovalisporum* and *C. raciborskii.* These species settled, proliferated and persist in the lake, due to their cellular and physiological capabilities, such as the formation of dormant cells (akinetes) that overwinter in the sediments, and can germinate when conditions are suitable. The abundance of *Microcystis* sp. in Lake Kinneret is mainly restricted to winter/spring, but frequent bloom events of these populations were recorded only during the last two decades. These winter populations presumably took advantage of nutrient supply from floods, overturn and internal sources, but were definitely affected by the presence/absence of other eukaryotic species, mainly the dinoflagellate, *Peridinium gatunense*. The increase in the abundance and diversity of cyanobacteria in Lake Kinneret was further supported by climatic and hydrological changes in the region. The establishment and persistence of Nostocales in Lake Kinneret may provide an example of the expansion of Nostocales worldwide.
